# Urolithiasis in Children: Medical Approach

**DOI:** 10.1016/j.pcl.2012.05.009

**Published:** 2012-08

**Authors:** Lawrence Copelovitch

**Affiliations:** Division of Nephrology, Department of Pediatrics, The Children's Hospital of Philadelphia, 34th Street and Civic Center Boulevard, Philadelphia, PA 19104, USA

**Keywords:** Urolithiasis, Nephrolithiasis, Renal calculi, Hypercalciuria, Hyperoxaluria, Hypocitraturia, Cystinuria, Children

## Abstract

Childhood urolithiasis is an evolving condition with an increasing incidence and prevalence over the last 2 decades. Over that time the underlying cause has shifted from predominantly infectious to metabolic in nature. This review describes the pathophysiology, underlying metabolic abnormalities, clinical presentation, evaluation, and management of childhood urolithiasis. A comprehensive metabolic evaluation is essential for all children with renal calculi, given the high rate of recurrence and the importance of excluding inherited progressive conditions.

**Key Points**

• The incidence and prevalence of childhood urolithiasis has been increasing over the last decade.
• The majority of renal calculi in children are comprised of either calcium oxalate or calcium phosphate and are often associated with a metabolic abnormality.
• Idiopathic hypercalciuria and hypocitraturia are the most frequently reported metabolic abnormalities.
• Given the high risk of recurrences in children with idiopathic hypercalciuria and hypocitraturia and the importance of excluding rare but treatable conditions such as primary hyperoxaluria and cystinuria a comprehensive metabolic evaluation is indicated in all children.

## Introduction

Urolithiasis is a fairly common disease in adults with an estimated prevalence of 3% to 5%.^1^ In economically developed countries, urolithiasis has been regarded as an uncommon condition in children. The estimated incidence in the United States from the 1950s to the 1970s is approximately 1% to 2% that of adults.^2,3^ More recent studies from the United States suggest an increase in the incidence and prevalence,^4,5^ with one study demonstrating a nearly 5-fold increase in the incidence in the last decade.^4^ Reports regarding gender predisposition have varied, with some studies suggesting equal prevalence and others indicating a greater risk among boys.^6^ Race and geography seem to play a vital role in the prevalence and cause of pediatric stone disease. In certain regions, such as Southeast Asia, the Middle East, India, and Pakistan, calculi are endemic. Calculi are particularly uncommon in children of African descent. The endemic calculi observed in developing nations are often confined to the bladder and comprise predominantly ammonium acid, urate, and uric acid, and seem to correlate with a decreased availability of dietary phosphates. In the United States, urolithiasis seems to be more common in Caucasian children from the Southeastern region. Over the last 3 decades the cause of childhood urolithiasis in the United Kingdom has shifted from predominantly infectious to metabolic in nature.^7^ Most calculi in the United States are found in the kidneys or ureters, comprise either calcium oxalate or calcium phosphate, and often associated with a metabolic abnormality.^8^

## Pathophysiology

Urolithiasis is associated with an identifiable metabolic abnormality in approximately 40% to 50% of children.^7–10^ The major metabolic abnormalities include: hypercalciuria, hyperoxaluria, hypocitraturia, cystinuria, and hyperuricosuria. Hypercalciuria or hypocitraturia are the most frequently reported abnormalities in children.^4,7^ In the United States approximately 40% to 65% of calculi comprises calcium oxalate, 14% to 30% of calcium phosphate, 10% to 20% of struvite, 5% to 10% of cystine, and 1% to 4% of uric acid.^8,11^ Rarely, stones may also comprise xanthine, or 2,8-dihydroxyadenine.

The initiation and growth of calculi requires the supersaturation of certain ions in the urine. The most important determinants of urine solubility and the likelihood of ion supersaturation (crystallization) are the total urine volume, the concentration of the stone-forming ions, the concentration of inhibitors of crystallization, the concentration of promoters of crystallization, and the urine pH. All types of calculi are less likely to form in dilute urine. Citrate, magnesium, pyrophosphate, certain glycosaminoglycans, nephrocalcin, and phytates all act to inhibit crystallization of calcium oxalate and calcium phosphate. Citrate acts as an inhibitor for the formation of calcium stones and binds to urinary calcium, thereby forming a soluble complex, which decreases the availability of free ionic calcium necessary for calcium oxalate or calcium phosphate crystallization. Citrate also acts as a direct inhibitor of calcium crystal aggregation and growth.^12,13^ Conversely, the presence of uric acid promotes calcium oxalate crystallization, which exemplifies the process of epitaxy, in which the crystal base of one material allows the growth of a second mineral that it is in the same crystalline orientation. Urine pH is important in that certain crystals such as cystine (pH <7.5) and uric acid (pH <6.0) are more likely to aggregate in acid urine whereas calcium phosphate (pH >6) is more likely to precipitate in alkaline urine. Calcium oxalate solubility is not appreciably affected by changes in urinary pH within the physiologic range.

Crystals in the urine usually form on the surface of a nidus that allows nucleation, growth, and aggregation of a stone particle at much lower concentrations than would be required otherwise. Any source of uroepithelial damage (eg, infection, foreign body, or Randall plaques) can serve as a nidus. Randall plaques comprise calcium phosphate crystals, which originate in the basement membrane in the thin loops of Henle. As the crystals aggregate they fuse into plaques in the interstitium and finally extrude through the uroepithelium of the renal papillae. Here they form a nidus and are thought to be critical in the formation of most cases of idiopathic calcium oxalate calculi. As a result, calcium oxalate calculi, either as monohydrate (whewellite) or as dihydrate (weddellite), are often admixed with small amounts of calcium phosphate, which form the initial nidus of the stone. Stones comprising predominantly calcium phosphate (brushite) are less common and seem to originate from plugging of the inner medullary collecting ducts.^14^

Genitourinary anomalies (hydronephrosis, duplex ureter, posterior uretheral valves, and bladder exstrophy) are found in approximately 30% of children with urolithiasis.^11^ Functional or anatomic obstruction predisposes children to stone formation by promoting stasis of urine and infection. Only 1% to 5% of children with urologic abnormalities develop calculi,^15^ suggesting a concomitant metabolic abnormality in patients with both urologic abnormalities and calculi. To emphasize this point, a study of 22 children with ureteropelvic junction obstruction and noninfectious calculi demonstrated that 15 (68%) had at least 1 concomitant metabolic abnormality, with hypercalciuria being the most common.^16^ Although infection is commonly associated with kidney stones, it is unlikely to be causative of non–struvite calculi. Although an important source of calculi in children, struvite stones will not be discussed further in this review because the only medical therapy centers on appropriate antibiotic treatment.

## Metabolic abnormalities

### Hypercalciuria

Hypercalciuria is found in approximately 30% to 50% of stone-forming children.^8,9^ Hypercalciuria is not a single entity but a condition associated with many causes (Box 1). The most common cause in children and adults is idiopathic hypercalciuria. Idiopathic hypercalciuria is defined as hypercalciuria that occurs in the absence of hypercalcemia in patients in whom no other cause can be identified. The gene (or genes) responsible for familial idiopathic hypercalciuria has not been identified, but appear to be transmitted in an autosomal dominant fashion with incomplete penetrance. Approximately 4% of asymptomatic healthy children demonstrate evidence of idiopathic hypercalciuria,^17^ and 40% to 50% of those children have a positive family history of urolithiasis.^18^ Hypercalciuria is formally defined as calcium excretion of greater than 4 mg/kg/d in children older than 2 years. In many children, a 24-hour urine collection is not practical and a urine calcium to creatinine ratio is used to estimate daily calcium excretion (Table 1). In school-aged children, a calcium to creatinine ratio of 0.2 mg/mg or less is considered normal, although higher values are reported in younger children.

When hypercalciuria is observed, several conditions must be excluded before establishing a diagnosis of idiopathic hypercalciuria. By definition the patient should be normocalcemic. In patients with hypercalcemic hypercalciuria, the possibility of hyperparathyroidism and hypervitaminosis D should be investigated and, when clinically indicated, a diagnosis of prolonged immobilization, sarcoidosis, malignancy, juvenile idiopathic arthritis, corticosteroid excess, adrenal insufficiency or William syndrome should be considered. Children with hypocalcemic hypercalciuria should be evaluated for hypoparathyroidism and autosomal, dominant hypocalcemic hypercalciuria (gain of function mutation in the calcium-sensing receptor). Although the vast majority of patients with normocalcemic hypercalciuria will ultimately be diagnosed with idiopathic hypercalciuria, other associated conditions, such as prematurity, diuretic exposure (furosemide and acetazolamide), anticonvulsant usage (topiramate and zonisamide), the ketogenic diet, Dent disease, Bartter syndrome, familial hypomagnesemia with hypercalciuria and nephrocalcinosis (FHHNC), distal renal tubular acidosis (dRTA), hereditary hypophosphatemic rickets with hypercalciuria (HHRH), and possibly medullary sponge kidney should be excluded and considered during the initial evaluation.

#### Genetic conditions associated with normocalcemic hypercalciuria

Dent disease is an X-linked inherited condition caused by a mutation in the *CLCN5* gene. The condition is characterized by low-molecular-weight proteinuria, nephrocalcinosis, hypercalciuria, nephrolithiasis, and chronic kidney disease. The clinical presentation is often insidious with many patients remaining asymptomatic throughout childhood; however, signs and symptoms of nephrocalcinosis and hypercalciuria are not uncommon in childhood. The defect is in proximal tubular function, and occasionally glucosuria, aminoaciduria, metabolic acidosis, and hypophosphatemia may all occur as part of an associated partial Fanconi syndrome. In a minority of patients, the Dent phenotype results from a mutation in the *OCRL* gene (Dent 2), which is also involved in the oculocerebrorenal syndrome of Lowe.

Bartter syndrome is an autosomal recessive condition characterized by renal salt wasting, hypokalemia, metabolic alkalosis, hypercalciuria, and normal serum magnesium levels. Children younger than 6 years typically present with salt craving, polyuria, dehydration, emesis, constipation, and failure to thrive. Severe polyhydramnios, prematurity, and occasionally sensorineural deafness are the hallmark features. Mutations in the *SLC12A, KCNJ1,* and *BSND* genes (Bartter syndrome type I, type II, and type IV, respectively) typically result in severe dysfunction of the thick ascending limb (TAL) of the loop of Henle in the neonatal period (neonatal Bartter syndrome). Mutations in the *ClCKB* gene (Bartter syndrome type III) usually cause milder TAL dysfunction and often present outside the neonatal period (classic Bartter syndrome).

FHHNC often presents in childhood with seizures or tetany caused by hypomagnesemia. Other clinical manifestations include frequent urinary tract infections (UTI), polyuria, polydipsia, failure to thrive, nephrolithiasis, and progressive renal failure.^19^ FHHNC is an autosomal recessive condition caused by mutations in either the *CLDN-16* or *CLDN-19* genes. Homozygous *CLDN*-16 or -19 mutations are associated with impaired tight junction integrity in the TAL, urinary magnesium and calcium wasting, and resultant hypomagnesemia. Patients usually develop the characteristic triad of hypomagnesemia, hypercalciuria, and nephrocalcinosis. Profound visual impairment characterized by macular coloboma, significant myopia, and horizontal nystagmus can been seen in association with *CLDN-19* mutations.^20^

Primary dRTA is an inherited condition characterized by systemic acidosis as a result of the inability of the distal tubule to adequately acidify the urine. Failure to thrive, polyuria, polydipsia, hypercalciuria, hypocitraturia, nephrocalcinosis, renal calculi, and hypokalemia are common presenting signs in infancy. Primary dRTA may be a dominant (*SLC4A1* gene) or a recessive condition (*ATP6V1B1* or *ATP6V0A4* genes). The inability to secrete H^+^ ions from the α-intercalated cells of the distal tubule is caused by either a defective vacuolar H^+^-ATPase (*ATP6V1B1* or *ATP6V0A4* genes) or a defective Cl^−^/HCO3^−^ anion exchanger-1 (*SLC4A1* gene). Sensorineural hearing loss may be found in patients with *ATP6V1B1* mutations.

HHRH is a rare, autosomal recessive disorder caused by mutations in the *SLC34A3* gene, resulting in loss-of-function of the type IIc sodium phosphate cotransporters of the proximal tubule. The decreased renal phosphate reabsorption can result in profound hypophosphatemia, normocalcemia, rickets, and bone pain. Hypercalciuria and nephrolithiasis are also commonly observed and may be the result of a hypophosphatemia-induced stimulation of 1,25-dihydroxyvitamin D synthesis. The increased synthesis purportedly causes increased gastrointestinal absorption of calcium and excessive urinary calcium losses in the face of normal serum calcium levels.^21^

### Hyperoxaluria

Oxalate is an end product of the metabolic pathways for glyoxylate and ascorbic acid and is primarily excreted by the kidneys. The vast majority (80%–85%) of daily urinary oxalate excretion is derived from normal metabolic homeostasis, and the remainder (10%–15%) is from dietary intake. Daily urine oxalate excretion is generally less than 50 mg/d/1.73 m^2^ of body surface area. The impracticality of performing 24-hour urine collections in very young patients requires the use of a random urine oxalate to creatinine ratio, which can be used to estimate oxalate excretion (see Table 1). Increased urinary oxalate excretion may be caused by an inherited metabolic disorder (primary hyperoxaluria [PH]) or, more commonly, as a secondary phenomenon caused by increased oxalate absorption or excessive intake of oxalate precursors.

#### PH

PH type I and II are relatively rare, autosomal recessive disorders of endogenous oxalate production. Overproduction of oxalate by the liver causes excessive urinary oxalate excretion with resultant nephrocalcinosis and nephrolithiasis. The calcium oxalate deposition results in progressive renal damage; however, the clinical presentation can vary from end-stage renal failure in the neonate to occasional stone passage into adulthood. Because of the clinical variability, the diagnosis is often overlooked and only realized after the loss of a transplanted kidney.^22^

PH type I is caused by mutations in the *AGXT* gene, which result in a functional defect of the hepatic peroxisomal enzyme alanine–glyoxylate aminotransferase (AGT). The deficit leads to accumulation of glyoxylate, glycolate, and oxalate in the urine.Pyridoxine is an essential cofactor for proper AGT activity and, rarely, profound vitamin B6 deficiency can mimic PH type I. PH type II is caused by mutations in the *GRHPR* gene with resultant deficient glyoxylate reductase–hydroxypyruvate reductase enzyme activity. Excessive amounts of oxalate and l-glyceric acid are excreted by the kidney.^23^ PH type II is somewhat milder compared with PH type I but is not benign. Recently, a third variant, PH type III has been described in 8 families with hyperoxaluria and mutations in the *DHDPSL* gene.^24^ The exact mechanism by which hyperoxaluria occurs in PH type III is yet to be fully elucidated.

#### Secondary hyperoxaluria

In secondary hyperoxaluria, there is either a dietary exposure to large amounts of oxalate (or oxalate precursors) or an underlying disorder that causes increased absorption of dietary oxalic acid from the intestinal tract. Gastrointestinal absorption varies inversely with dietary calcium intake, and, as a result, calcium-deficient diets may increase oxalate absorption and hyperoxaluria.^25^ Oxalate is a byproduct of ascorbic acid metabolism, and high doses of vitamin C have also been associated with hyperoxaluria. Increased dietary absorption is usually characterized by fat malabsorption or a chronic diarrheal disorder. Among secondary causes of hyperoxaluria, those attributable to gastrointestinal disease are inflammatory bowel disease, celiac disease, exocrine pancreatic insufficiency (cystic fibrosis), biliary tract disease, and small bowel resection or short bowel syndrome. The pathogenesis in these conditions results from the presence of free fatty acids that bind calcium in the intestinal lumen resulting in more unbound oxalate, which is free to be absorbed.

### Hypocitraturia

Citrate is normally present in the urine and regulated through a process of both absorption and metabolism at the level of the proximal tubule. Hypocitraturia is generally defined as a citrate to creatinine ratio of less than 180 mg/gm in men and less than 300 mg/gm in women on a 24-hour collection (see Table 1). Intracellular acidosis of the proximal tubule, caused by either metabolic acidosis or hypokalemia results in an increased citrate absorption in the proximal tubule and resultant hypocitraturia. As a result, the ketogenic diet, certain medications (topiramate, zonisamide, and acetazolamide), dRTA, and chronic diarrhea are commonly associated with hypocitraturia. Given that an incomplete dRTA can occur in the absence of an overt systemic acidosis or hypokalemia, the condition can often be overlooked in the face of hypocitraturia if provocative acid-load testing is not readily available. Despite these known associations, most cases of hypocitraturia are idiopathic although a diet rich in animal protein and low in vegetable fiber and potassium seems to promote lower citrate excretion.^26,27^

### Cystinuria

Cystinuria is an autosomal recessive disorder caused by mutations in either the *SLC3A1* or the *SLC7A9* genes, resulting in a disordered amino acid transport in the proximal tubule,^28^ Cystinuria is characterized by urinary hyperexcretion of cystine and the dibasic amino acids lysine, ornithine, and arginine. Normal individuals excrete less than 50–60 mg of cystine/d/1.73 m^2^ of body surface area, whereas patients who are homozygous for cystinuria often excrete greater than 400 mg/d/1.73 m^2^ of body surface area.^7^ Patients typically present with renal colic and urolithiasis in the second or third decade of life; however, they may present as early as infancy with staghorn calculi. The poor solubility of cystine in the urine causes precipitation in the collecting system, which, if left untreated, usually results in recurrent episodes of calculi and long-term risk for renal failure. Associated UTI's are common, and combined cystine and struvite calculi have been observed.^29^

In cystinuria, the disordered cystine transport primarily results from dysfunction of the heteromeric amino acid transporter (rBAT/b^0,+^AT), comprising heavy (rBAT) and light (b^0,+^AT) subunits. Cystinuria was originally classified into type I and non–type I (types II and III) based on the urinary cystine concentration pattern of obligate heterozygotes and the presumed mode of inheritance. Type I follows the classic autosomal recessive inheritance with heterozygotes showing normal cystine excretion. In contrast, non–type I (type II and III) heterozygotes demonstrate moderate or high excretion of urinary cystine. Types II and III differ in that type III homozygotes show a nearly normal increase in cystine plasma levels after oral cystine administration.^30^ It is now clear that homozygous mutations in the *SLC3A1* gene, which encodes rBAT is associated with type I cystinuira, and homozygous mutations in the *SLC7A9* gene, which encodes b^0,+^AT accounts for most cases of type II and III. A more recent classification system has been developed, which designates patients who are homozygous for the *SLC3A1* mutations as cystinuria type A, patients who are homozygous for the *SLC7A9* mutations as type B, and those who have a mutation in both the *SLC3A1* and *SLC7A9* genes as type AB.^31^

### Hyperuricosuria

Uric acid excretion is greater in children than in adults, with the highest urinary fractional excretion (Fe) found in neonates (Fe 30%–50%) and levels reaching adult values (Fe 8%–12%) in adolescence.^32^ Hyperuricosuria is defined as uric acid excretion of greater than 815 mg/d/1.73 m^2^ of body surface area. When adjusted for glomerular filtration rate (GFR), relative uric acid excretion is fairly constant after 2 years of age (see Table 1). In children who are not yet trained to use toilet but older than of 2 years, hyperuricosuria can be defined as greater than 0.56 mg/dL of GFR on a spot urine collection. This value may be calculated using Equation 1:

(1) Urineuricacid(mg/dL)×Plasmacreatinine(mg/dL)/Urinecreatinine(mg/dL)

Hyperuricosuria in the setting of low urinary pH is the greatest risk factor for uric acid stone formation. Hyperuricosuria associated with significant hyperuricemia is usually associated with inherited disorders of purine metabolism (see section on Inherited disorders of purine metabolism), lymphoproliferative disorders, and polycythemia. Rarely, a condition known as hereditary renal hypouricemia characterized by low serum uric acid, hyperuricosuria, nephrolithiasis, and exercise-induced acute renal failure has been observed. Mutations in either the *SLC22A12* or the *SLC2A9* genes, both of which encode urate transporters expressed in the proximal tubule, are known to be causative.^28^ Other causes of hyperuricosuria include excessive purine intake (animal protein, anchovies, and mussels), hemolysis, uricosuric medications (probenecid, salicylates, and losartan), cyanotic congenital heart disease, melamine toxicity, and idiopathic (familial). There is also a phenomenon primarily observed in adults called hyperuricosuric calcium oxalate urolithiasis in which hyperuricosuria seems to be the principle contributor to the development of calcium oxalate stones with either no or minimal uric acid content (epitaxy).

#### Inherited disorders of purine metabolism

Phosphoribosyl pyrophosphate synthetase superactivity (PRPSS) is an X-linked condition caused by mutations in the *PRPS1* gene. The overactive PRPSS is associated with excessive purine production. The subsequent purine degradation results in hyperuricemia, gout, hyperuricosuria, and uric acid nephrolithiasis. Some affected individuals have neurodevelopmental abnormalities, particularly sensorineural deafness.^33^ Hypoxanthine-guanine phosphoribosyl transferase (HPRT) deficiency is an X-linked inborn error of purine metabolism caused by mutations in the *HPRT1* gene associated with overproduction of uric acid. Complete deficiency of HPRT activity is associated with the Lesch-Nyhan syndrome, characterized by mental retardation, spastic cerebral palsy, choreoathetosis, uric acid calculi, and self-injurious behavior. Children with partial HPRT deficiency can be phenotypically similar to patients with complete deficiencies or may have more subtle or mild neurologic symptoms. Renal stones, uric acid nephropathy, renal obstruction, or gout may be the first presenting signs of the disease.^28^

## Clinical presentation

The classic adult presentation of acute, severe flank pain, which radiates to the groin is uncommon in children, particularly in children younger than 5 years. Although adolescents present similarly to adult patients, younger children have varied presentations including nonspecific pain localized to the abdomen, flank, or pelvis. In infants, symptoms of stones may be confused with colic pain. Macroscopic or microscopic hematuria can occur in up to 90% of children with urolithiasis.^34^ Ureteral stones are much more likely to cause obstruction that leads to pain. Renal stones may be found incidentally and remain present for years without causing symptoms. Approximately 10% of calculi can present with dysuria and urinary frequency and are usually localized to the lower urinary tract. UTI may also complicate nephrolithiasis, although pyuria may also be present without bacteriuria or infection. Rarely, a urethral stone can present with acute urinary obstruction.^11,35^

## Medical history and physical examination

Obtaining a thorough medical history followed by careful examination is essential for establishing an accurate diagnosis. Information pertaining to a family history of calculi, hematuria, and renal failure can be essential in identifying those patients at highest risk for inherited metabolic or genetic conditions (eg, cystinuria, primary hyperoxaluria, and Dent disease). A focused dietary history with special emphasis on fluid and salt intake, vitamin (C, D) mineral supplementation, and special diets (eg, ketogenic diet) is indicated in every patient. Eliciting a detailed medication history with special emphasis on corticosteroids, diuretics (furosemide and acetazolamide), protease inhibitors (indinavir), and anticonvulsants (topiramate and zonisamide) can be instructive. Children with a history of prematurity, urinary tract abnormalities, UTIs, intestinal malabsorption (eg, Crohn's disease, bowel resection, and cystic fibrosis), and prolonged immobility are all at special risk for calculi formation. Detailed physical examination of the child for dysmorphic features (William syndrome), rickets (Dent disease and HHRH), tetany (FHHNC and autosomal dominant hypocalcemic hypercalciuria), and gout (HPRT deficiency, PRPSS) can be helpful.

## Evaluation

### Imaging

The first step involved in the evaluation of urolithiasis is detection of the calculus. The sensitivity of plain abdominal radiography in the detection of calculi is approximately 45% to 58%; although many stones are radiopaque, radiography alone is insufficient in the evaluation of a patient with suspected urolithiasis.^36^ In addition, calculi comprising uric acid, cystine, xanthine, or indinavir are usually radiolucent. Ultrasonography (US) has the ability to detect 90% of calculi confined to the kidney; however, the sensitivity for detecting ureteral calculi and smaller calculi (<5 mm) is poor.^5^ Nonetheless, because radiation exposure is not without risk, US remains the initial study of choice in the assessment of calculi in children. Noncontrast computed tomography remains the gold standard and is indicated in children with persistent symptoms of urolithiasis and a nondiagnostic US. In patients with hypercalciuria in whom medullary sponge kidney is suspected, an intravenous pyelogram can be considered.

### Metabolic Investigations

When urinary calculi develop during childhood, the risk of life-long stone formation is significant, with approximately 16% to 20% having recurrences within 3 to 13 years.^10,37^ Furthermore, children with an identifiable metabolic abnormality have an up to 5-fold increased risk of having a recurrence as compared with children with no identifiable metabolic disorder.^10^ As a result, all children should undergo a comprehensive initial evaluation. Whenever possible, analysis should begin with an infrared spectroscopy or radiograph diffraction analysis of a passed stone. If a cystine or struvite stone is found, the analysis will be diagnostic.

Serum and urine studies should be obtained in patients in whom stone analysis could not be performed or for those with either calcium or uric acid-based stones. A serum creatinine level is essential to evaluate for possible acute kidney injury or chronic kidney disease. Serum calcium, phosphorous, bicarbonate, magnesium, and uric acid levels are effective in screening for hypercalcemia- and hypocalcemia-associated calculi (discussed earlier), hyperuricemia, HHRH, Bartter syndrome, dRTA, and FHHNC. Unlike in adults, primary hyperparathyroidism is rare in children and an intact parathyroid hormone level is not an essential part of the initial evaluation unless there is evidence of hypercalcemia and hypophosphatemia. A 25-hydroxyvitamin D level should be evaluated in all patients with hypercalcemia. A spot urine beta-2 microglobulin (low-molecular-weight protein) is a useful screening test for Dent disease and should be considered in men and possibly carrier women if there are recurrent calcium-based calculi in the setting of proteinuria or a family history of renal failure, focal segmental glomerulosclerosis, or recurrent calculi.

A 24-hour urine collection should be analyzed for calcium, oxalate, uric acid, sodium, citrate, creatinine levels, volume, pH, and cystine (cyanide-nitroprusside screening test). Results must be evaluated with respect to weight, body surface area, and creatinine level to be properly interpreted in children. Urine creatinine excretion (normal 15–25 mg/kg/d) is useful in assessing the adequacy of the urine collection. Supersaturations for calcium oxalate, calcium phosphate, and uric acid can be calculated from computer models based on the results of the urine collection. There is ongoing controversy as to whether a single 24-hour urine collection at the time of diagnosis is sufficient for proper evaluation^38^ or whether 2 separate collections yield a greater number of specific diagnoses.^39^ Several commercial companies, including Litholink, Mission, Dianon, and Urocor offer these 24-hour urine stone chemistry profiles. Although less precise, when children are not yet trained to use toilet, the evaluation may be performed by measuring the ratio of calcium, uric acid, citrate, and oxalate levels to creatinine level in a random urine sample. Repeat urine testing should be performed several weeks to months after a change in diet or after the initiation of a medication. Microscopic urinalysis for crystalluria is generally not diagnostic unless hexagonal crystals (cystine) or coffin lid–shaped triple phosphate crystals (struvite) are observed.

## Medical management

### Acute Management

The first goal of medical management should be directed toward control of the acute complications. Pain associated with the passage of a stone is often severe and should be treated promptly with narcotic analgesics (morphine sulfate) and/or nonsteroidal antiinflammatory drugs (Ketorolac). If the patient is vomiting or unable to drink, parenteral hydration should be used to maintain a high urine flow rate. In the absence of oligoanuric renal failure or a complete obstruction, an intravenous infusion rate of 1.5 to 2 times maintenance is recommended. Agents that may promote the passage of stones and reduce symptoms (medical expulsive therapy), such as alpha-adrenergic blockers (tamsulosin) and calcium-channel blockers (nifedipine), have shown promising results in adults with distal ureteral calculi.^40^ Although studies in children are limited, 1 prospective study showed that in children with distal ureteral calculi who were treated with tamsulosin, there was a greater stone expulsion rate and decreased time to stone expulsion when compared with controls.^41^ Urine should be strained for several days to recover any gravel or calculi passed for analysis. Because UTIs often present concomitantly in children with calculi, a urine culture should be obtained and empiric antibiotic therapy initiated if a UTI is suspected.

### Preventative Measures

#### Fluid

Fluid intake is a critical component of stone prevention by effectively reducing the concentration of lithogenic factors, including calcium, oxalate, uric acid, and cystine. Although high daily fluid intake reduces the risk of recurrent stone formation,^42^ the exact prescription is unknown. Most clinicians recommend intake at least equal to calculated maintenance rates in children and no less than 2 to 2.5 L in adolescents and adults. Even higher fluid intake levels (1.5–2 L/m^2^) may be recommended for children with cystinuria or PH. Increased intake requirements may be required during periods of increased insensible water loss. Regarding fluids other than water, reports suggest that fluids that increase urinary pH and citrate excretion such as orange juice, lemonade, and black currant juice, as well as those that increase urinary volume such as coffee, tea, beer, and wine, reduce the risk of calcium stone formation.^43^ Conversely, grapefruit juices seem to increase the risk of calcium-based stones.^43,44^ Whether cola drinks increase lithogenic potential or not remains controversial.^43,44^

#### Sodium

The association between sodium intake and calcium stone formation has been reported but has not been confirmed in all studies.^44^ Increased sodium intake is known to promote calciuria by competing for reabsorption at the level of the renal tubules. A low salt diet corresponding to less than 2 to 3 mEq/kg/d in children or less than 2.4 g/d in adolescents or adults is generally recommended for patients with hypercalciuria or calcium-containing stones. A low salt diet may also reduce urinary cystine excretion in patients with cystinuria.

#### Calcium

Until recently, higher calcium intake was thought to increase the risk of stone formation; however, there is substantial evidence now that a higher calcium containing diet is associated with a reduced risk of stone formation.^45^ A potential mechanism that might explain this paradox is that higher calcium intake effectively binds dietary oxalate in the gut, thereby reducing intestinal absorption and eventual urinary oxalate excretion. The current recommendation for stone formers is to curtail excess calcium intake, but calcium restriction is not recommended, in part, because of the long-term risk of osteoporosis. Excess consumption of vitamin D with or without calcium supplements can also induce excessive urinary calcium excretion.

#### Animal protein

There is compelling evidence for a role of dietary animal proteins (meat, fish, and poultry) in calcium oxalate stone formation. The metabolism of sulfur-containing amino acids in animal meat generates an acid load in the form of sulfuric acid. As a result, excessive dietary animal protein intake causes increased urinary calcium excretion and reduced urinary citrate excretion and pH. Vegetable and dairy protein sources do not seem to carry the same lithogenic potential. The consumption of excessive amounts of dietary animal protein also results in increased purine intake, increased uric acid production, and may contribute to both uricosuria and more acidic urine. In patients with cystinuria, there is little evidence to support the dietary restriction of proteins high in cystine content; however, reducing animal protein intake might be helpful by increasing urinary pH. Children with calculi are recommended not to eat excessive amounts of protein but should aim for 100% of the daily recommended allowance for age to supply adequate substrate for growth and nutrition.

#### Oxalate

The role of dietary oxalate in stone formation is controversial because only approximately 10% to 20% of urinary oxalate excretion is derived from the diet. As a precautionary measure, most clinicians recommend limiting dietary oxalate ingestion in calcium oxalate stone formers who demonstrate evidence of hyperoxaluria. Foods that contain high levels of oxalate include certain nuts (almonds, peanuts, cashews, walnuts, and pecans), spinach, soy beans, tofu, rhubarb, beets, sweet potatoes, wheat bran, okra, parsley, chives, black raspberries, star fruit, green tea, and chocolate. Vitamin C supplements have been associated with increased risk of calcium oxalate stone formation because oxalate is a byproduct of ascorbic acid metabolism and therefore, these supplements should be discontinued in calcium oxalate stone formers with hyperoxaluria.^46^

#### Potassium/citrate

Potassium-rich foods such as fruits and vegetables usually contain large amounts of citrate, which are protective against the formation of calcium oxalate stones. In many studies, a diet high in potassium is protective against urolithiasis.^45^ In addition, a potassium-restricted diet can cause increased urinary calcium excretion and overt hypokalemia, leading to hypocitraturia. One recent study suggests that chronically low potassium intake in the absence of overt hypokalemia may also result in low urinary potassium and citrate levels.^47^ As a result, a diet containing potassium-rich fruits and vegetables can theoretically increase urinary citrate excretion directly because of the citrate content found in those foods and indirectly through the dietary potassium content.

#### Others

Magnesium complexes with oxalate and may prevent enteric oxalate absorption as well as decrease calcium oxalate supersaturation in the urine. In some studies, higher dietary magnesium has been associated with a lower risk of stone formation in men,^46^ and supplementation may be helpful in the treatment of children with secondary hyperoxaluria. Carbohydrate ingestion has been associated with hypercalciuria, and sucrose ingestion has been found to be associated with urolithiasis.^45^ Phyate, a dietary factor found in many high fiber-containing foods (cereals, legumes, vegetables, and nuts), seems to bind calcium avidly and may inhibit the formation of calcium oxalate stones.

### Medications

Pharmacotherapy is recommended for children in whom fluid and dietary therapy is ineffective in controlling the formation of stones, or for those with primary hyperoxaluria, cystinuria, or a known genetic condition associated with normocalcemic hypercalciuria (see previous section on Genetic conditions associated with normocalcemic hypercalciuria).

#### Diuretics

A thiazide diuretic is often required for children with hypercalciuria who do not respond to a restricted sodium diet. The usual recommendation is hydrochlorothiazide 1 to 2 mg/kg/d (adult 25–100 mg/d). Amiloride can be added for its potassium-sparing effect as well as for its ability to independently reduce calcium excretion. Alternatively, potassium citrate could be provided to mitigate the effects of potassium depletion. Thiazide diuretics have also been used in an attempt to reduce calcium excretion in patients with Dent disease, FHHNC, and PH.

#### Alkali agents

Treatment with either potassium citrate (2–4 mEq/kg/d, adults 30–90 mEq/d)^48^ or potassium-magnesium citrate^49^ has been shown to reduce the recurrence of calcium oxalate stone formation in patients with low or normal citrate excretion. Sodium citrate is generally considered less ideal because it is associated with increased sodium delivery to the nephron. Treatment is considered safe with only minor gastrointestinal side effects; however, one potential concern is that over-treatment with alkali may increase the risk of calcium phosphate stone formation by increasing the urinary pH to greater than 6.5, thereby decreasing the calcium phosphate supersaturation product. Potassium citrate is also used to alkalinize the urine in patients with Dent disease, FHHNC, dRTA, uric acid lithiasis (goal of urine pH >6.5), cystinuria (goal of urine pH >7), and hyperoxaluria.

#### Thiol-containing agents

These agents are used exclusively for patients with cystinuria in whom fluid and dietary modifications as well as urinary alkalinization are ineffective in preventing stone recurrences or dissolving preexisting stones. The 2 most common agents are d-penicillamine and α-mercaptopropionylglycine (tiopronin). Cystine is formed as a dimer of cysteine and these agents work by reducing the disulfide bond that bridges the 2 molecules of cysteine. The thiol group combines with cysteine to form a more soluble cysteine-drug product combination, which is be excreted. d-penicillamine has a large number of adverse side effects, including febrile reactions, gastrointestinal discomfort, liver dysfunction, impaired taste, bone marrow suppression, trace metal deficiencies, membranous glomerulopathy, myasthenia gravis, and skin eruptions (elastosis perforans serpiginosa). The incidence of adverse effects for α-mercaptopropionylglycine is similar but may be slightly less. Monitoring of liver enzymes, complete blood count, urinalysis, and copper and zinc levels should be performed regularly. Special assays (solid-phase assay or high performance liquid chromatography) can readily distinguish between urinary cystine and cysteine-drug complexes and may help in guiding long-term medical therapy.

#### Allopurinol

The mainstay of therapy for most children with uric acid calculi is a combination of high urine flow rate and alkalinization of the urine. Allopurinol (4–10 mg/kg/d, adult maximum 300 mg/d) is indicated conditions in which there is both hyperuricemia and hyperuricosuria, such as PRPSS or HPRT deficiency. Inhibition of xanthine dehydrogenase by allopurinol may lead to the accumulation and urinary excretion of xanthine. Rarely, a secondary xanthinuria with xanthine calculi is observed in children on long-term therapy. Allopurinol may also be the agent of choice for treating hyperuricosuric calcium oxalate urolithiasis if there is no concomitant evidence of hypercalciuria, hyperoxaluria, or hypocitraturia.^50^

#### Pyridoxine

Pyridoxine is an important cofactor of AGT. Approximately 10% to 30% of children with PH type I are pyridoxine sensitive (>30% reduction of urinary oxalate excretion). In particular, patients who are homozygous for Gly170Arg or Phe152Ile mutations are more likely to respond and have preserved renal function over time with adequate treatment.^42^ In patients with suspected PH type I, treatment should be initiated (2–5 mg/kg/d) and titrated upward (8–10 mg/kg/d) until a diagnosis can be made and response assessed. Large doses of pyridoxine have been known to induce sensory neuropathies. There is currently no evidence to suggest that pyridoxine supplementation is beneficial in the treatment of other forms of hyperoxaluria unless a true pyridoxine deficiency is present.

## Figures and Tables

**Table 1:** Normal values for urinary solute excretion

Metabolite	Age	Random (mg/mg)	24-h (All Ages)
Calcium	0–6 mo 7–12 mo >24 mo	<0.8 <0.6 <0.21	<4 mg/kg/d
Oxalate	0–6 mo 7–24 mo 2–5 y 5–14 y >16 y	<0.26 <0.11 <0.08 <0.06 <0.03	<50 mg/1.73 m^2^
Citrate	0–5 y >5 y	>0.2–0.42	>180 mg/gm Males, >300 mg/gm Females
Cystine	>6 mo	<0.075	<50 mg/1.73 m^2^
Uric acid	>2 y	0.56 mg/dL GFR^a^	<815 mg/1.73 m^2^

a Equation 1: Urine uric acid (mg/dL) × Plasma creatinine (mg/dL)/Urine creatinine (mg/dL).

## Box 1 Clinical disorders associated with hypercalciuria

**Hypercalcemia**
Hyperparathyroidism
Hypervitaminosis D
Immobilization
Sarcoidosis
Malignancy
Juvenile idiopathic arthritis
Corticosteroid excess
Adrenal insufficiency
Williams syndrome
Idiopathic hypercalcemia of infancy

**Hypocalcemia**
Hypoparathyroidism
Autosomal dominant hypocalcemic hypercalciuria

**Normocalcemia**
*Acquired*
Prematurity
Furosemide
Topiramate
Ketogenic diet
*Genetic*
Idiopathic
Dent disease
Bartter syndrome
Familial hypomagnesemia with hypercalciuria and nephrocalcinosis
Primary distal renal tubular acidosis (dRTA)
Hereditary hypophosphatemic rickets with hypercalciuria
*Other*
Medullary sponge kidney
Secondary dRTA
Glycogen-storage disease type I
